# Supplementary material for: Intermittent dietary methionine deprivation facilitates tumoral ferroptosis and synergizes with checkpoint blockade
Source: Nat Commun. 2023 Aug 8;14:4758. doi: 10.1038/s41467-023-40518-0 (PMC10409767; doi:10.1038/s41467-023-40518-0)
Supplement: Supplementary file 1 — Supplementary Information [file 41467_2023_40518_MOESM1_ESM.pdf]

SUPPLEMENTARY INFORMATION

Intermittent dietary methionine deprivation facilitates tumoral ferroptosis and synergizes with checkpoint blockade

Ying Xue[1,#], Fujia Lu[1,#], Zhenzhen Chang[1], Jing Li[1], Yuan Gao[2], Jie Zhou[1], Ying Luo[3], Yongfeng Lai[1], Siyuan Cao[1], Xiaoxiao Li[1], Yuhan Zhou[1], Yan Li[1], Zheng Tan[1], Xiang Cheng[4], Xiong Li[5], Jing Chen[6], Weimin Wang[1,7,8,*]

[1]Department of Immunology, School of Basic Medicine, Tongji Medical College, Huazhong University of Science and Technology, Wuhan, China.
[2]Department of Oncology, Tongji Hospital, Tongji Medical College, Huazhong University of Science and Technology, Wuhan, China.
[3]Department of Laboratory Medicine, Tongji Hospital, Tongji Medical College, Huazhong University of Science and Technology, Wuhan, China.
[4]Department of Cardiology, Union Hospital, Tongji Medical College, Huazhong University of Science and Technology, Wuhan, China.
[5]Department of Gynecology & Obstetrics, the Central Hospital of Wuhan, Tongji Medical College, Huazhong University of Science and Technology, Wuhan, China.
[6]Cancer Center, Union Hospital, Tongji Medical College, Huazhong University of Science and Technology, Wuhan, China.
[7]Cell Architecture Research Institute, Huazhong University of Science and Technology, Wuhan, China.
[8]Key Laboratory of Organ Transplantation, Ministry of Education; NHC Key Laboratory of Organ Transplantation; Key Laboratory of Organ Transplantation, Chinese Academy of Medical Sciences, Wuhan, China.

[#] These authors contributed equally.

* Correspondence: Weimin Wang, email: weiminw@hust.edu.cn

Supplementary Figures

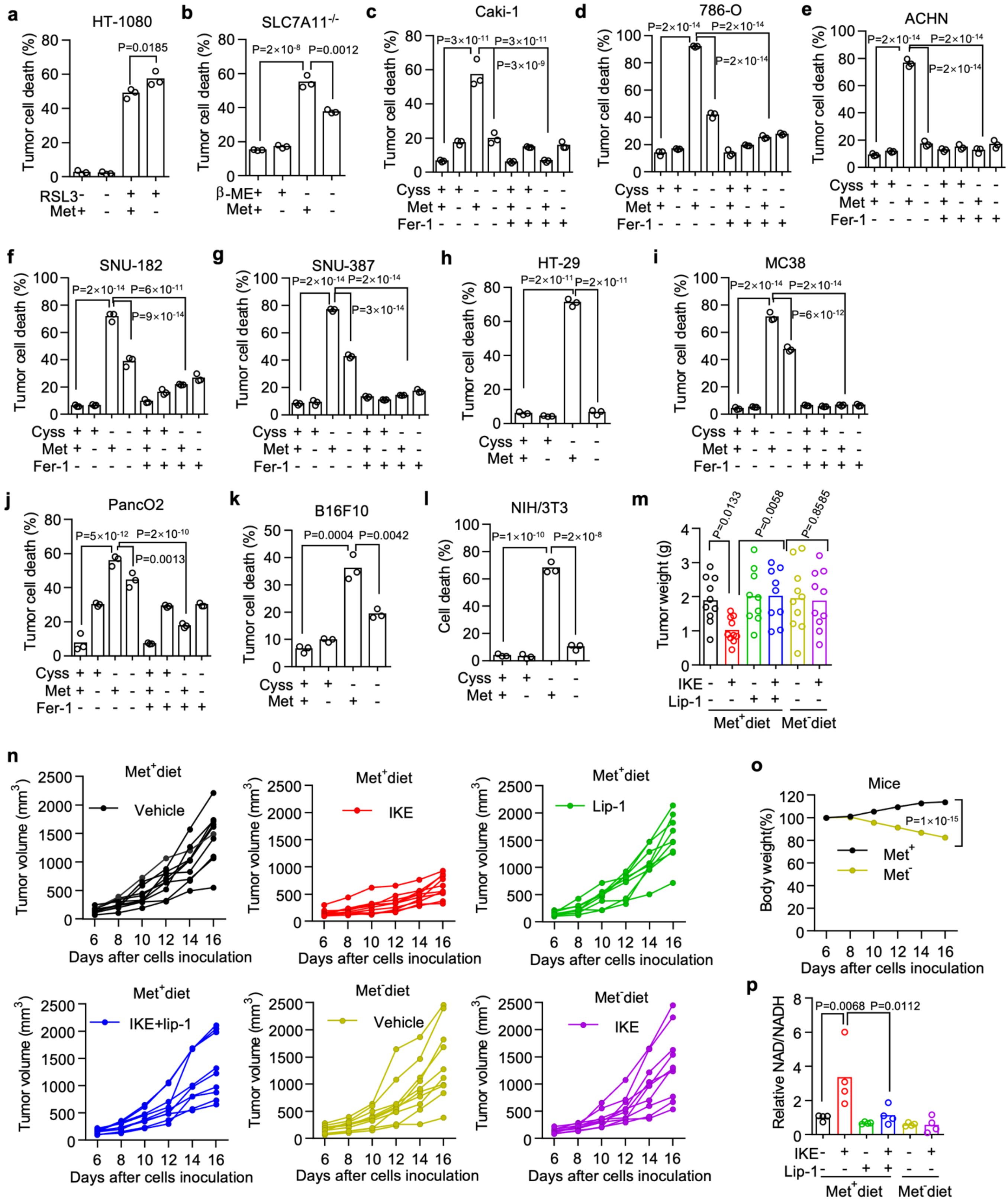

Supplementary Figure 1. Prolonged methionine deprivation blocks ferroptosis induced by cystine deprivation or system xc- inhibition, but not by GPX4 inhibition.

a, Cell death of HT-1080 cells cultured in methionine-free medium and treated with RSL3 (0.5 μM) for 16 h. n = 3 biological replicates (one-way ANOVA).

b, Cell death of SLC7A11 deficient ($SLC7A11^{-/-}$) HT-1080 cells treated with β-ME (50 μM) withdrawal plus methionine co-withdrawal for 24 h.

c- l, Effect of methionine co-deprivation on cystine deprivation-induced ferroptosis in various human and mouse tumor cell lines. Cell death was detected at different time points: Caki-1 (**c**, 38h), 786-O (**d**, 26h), ACHN (**e**, 32h), SNU-182 (**f**, 37h), SNU387 (**g**, 38h), HT-29 (**h**, 48h), MC38 (**i**, 22h), PancO2 (**j**, 26h), B16F10 (**k**, 22h) and NIH/3T3 (**l**, 18h). Fer-1 (10 μM) was added to confirm the cell death is ferroptosis. n = 3 biological replicates (one-way ANOVA).

m- p, Hepa1-6 tumor-bearing mice were fed with control (Met^{+}) or methionine free (Met^{-}) diet and treated with IKE (40 mg/kg), liproxstatin-1 (Lip-1, 10mg/kg) or their combination (n = 9 or 10 mice per group). Subcutaneous tumors were surgically removed and their weights were measured at the end point (**m**). Individual tumor growth was monitored over time (**n**). Mice body weights were monitored once switch to Met^{-} diet (**o**). NAD/NADH ratio in isolated tumor tissues was quantified (**p**). Data were presented as mean ± s.e.m. and P value is determined by one-way ANOVA (**m, p**) or two-way ANOVA (**o**).

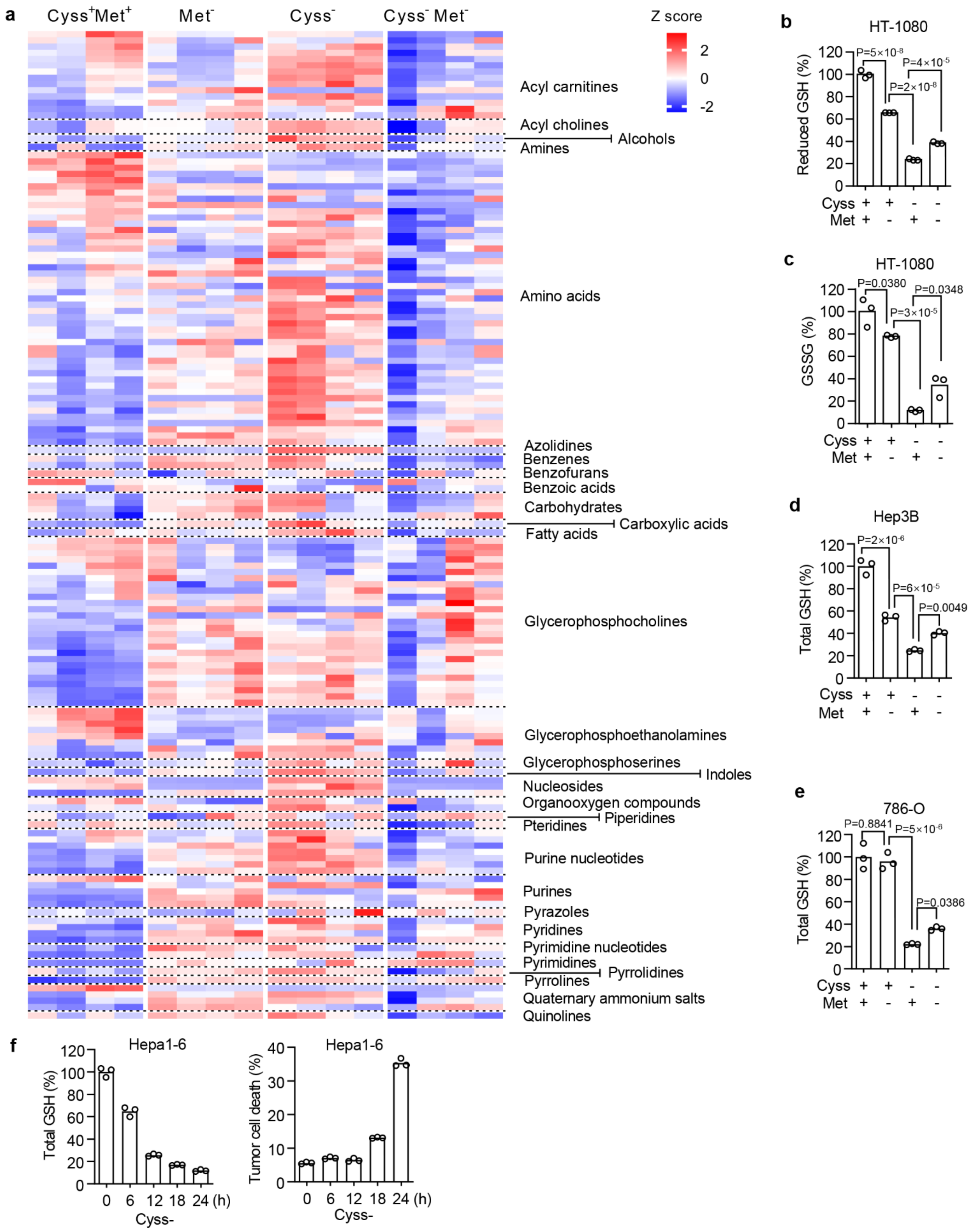
a
Cyss+Met+
Met-
Cyss-
Cyss- Met-
Z score
2
0
-2
Acyl carnitines
Acyl cholines
Alcohols
Amines
Amino acids
Azolidines
Benzenes
Benzofurans
Benzoic acids
Carbohydrates
Carboxylic acids
Fatty acids
Glycerophosphocholines
Glycerophosphoethanolamines
Glycerophosphoserines
Indoles
Nucleosides
Organooxygen compounds
Piperidines
Pteridines
Purine nucleotides
Purines
Pyrazoles
Pyridines
Pyrimidine nucleotides
Pyrimidines
Pyrrolidines
Pyrrolines
Quaternary ammonium salts
Quinolines
b
HT-1080
Reduced GSH (%)
P=5×10-8
P=4×10-5
P=2×10-8
Cyss + + - -
Met + - + -
c
HT-1080
GSSG (%)
P=0.0380
P=0.0348
P=3×10-5
Cyss + + - -
Met + - + -
d
Hep3B
Total GSH (%)
P=2×10-6
P=6×10-5
P=0.0049
Cyss + + - -
Met + - + -
e
786-O
Total GSH (%)
P=0.8841
P=5×10-6
P=0.0386
Cyss + + - -
Met + - + -
f
Hepa1-6
Total GSH (%)
0 6 12 18 24 (h)
Cyss-
Hepa1-6
Tumor cell death (%)
0 6 12 18 24 (h)
Cyss-

Supplementary Figure 2. Prolonged methionine deprivation impairs substantial GSH depletion induced by cystine deprivation.

a, Heatmap of metabolites belonging to different classes that are identified by LC-MS in HT-1080 cells. Cells were treated by cystine or methionine deprivation or their co-deprivation. n = 4 biological replicates.

b, c, Reduced GSH (**b**) and oxidized GSSG (**c**) content in HT-1080 cells cultured in cystine or methionine free or their double free medium for 6 h.

d, e, Total GSH content in Hep3B (**d**) or 786-O (**e**) cells cultured in cystine or methionine free or their double free medium for 8 h.

f, Total GSH content and time points matched cell death of Hepa1-6 cells upon cystine deprivation.

n = 3 biological replicates and P values are determined using one-way ANOVA (**b-e**).

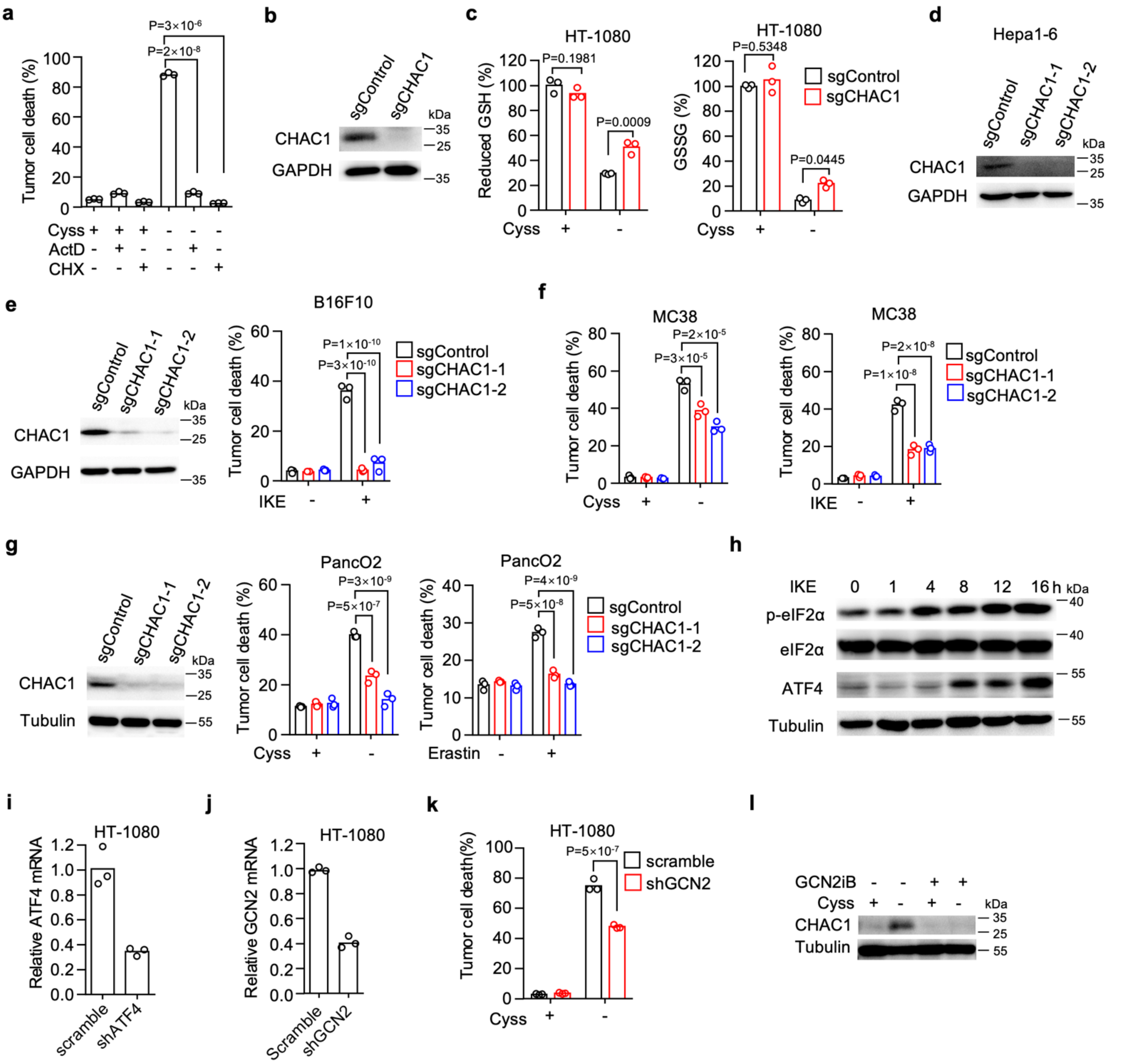

Supplementary Figure 3. CHAC1 induction by cystine deprivation is mediated through GCN2-ATF4 pathway.

a, Cell death of HT-1080 cells treated with cystine deprivation in the presence of Actinomycin D (ActD, 5 μM) or CHX (1μM) for 18 h.

b, c, Immunoblot of human CHAC1 in HT-1080 cells expressing sgControl or sgCHAC1 (**b**). Reduced GSH or oxidized GSSG contents in these cells under cystine deprivation for 8 h were measured (**c**).

d, Immunoblot of murine CHAC1 in Hepa1-6 cells expressing sgControl or two different sgCHAC1.

e, CHAC1 immunoblot in B16F10 cells expressing sgControl or two sgCHAC1. Cell deaths of these

cells treated with IKE (1 μM) for 28 h were quantified.
f, Cell deaths of MC38 cells expressing sgControl or two different sgCHAC1 in response to cystine deprivation for 28 h or IKE (2 μM) treatment for 30 h.
g, Immunoblot of murine CHAC1 in PancO2 cells expressing sgControl or sgCHAC1. Cell deaths of these cells under cystine deprivation for 29 h or erastin (10 μM) treatment for 29 h.
h, Immunoblots of phosphorylated eIF2α (p-eIF2α), total eIF2α and ATF4 in HT-1080 cells treated by 0.5 μM IKE for indicated time points. Tubulin serves as loading control.
i, Relative ATF4 mRNA level in scramble or ATF4 shRNA (shATF4) expressing HT-1080 cells.
j, Relative GCN2 mRNA level in scramble or GCN2 shRNA (shGCN2) expressing HT-1080 cells.
k, Cell death of scramble or shGCN2 expressing HT-1080 in response to cystine deprivation for 15 h.
l, Immunoblot of CHAC1 in HT-1080 cells treated by cystine deprivation plus GCN2 inhibitor (GCN2iB, 1 μM) for 8 h.

The experiment was repeated twice with similar results (**d**, **h** and **l**). n = 3 biological replicates and P values are determined by one-way ANOVA (**a**) or two-way ANOVA (**c, e, f, g** and **k**).

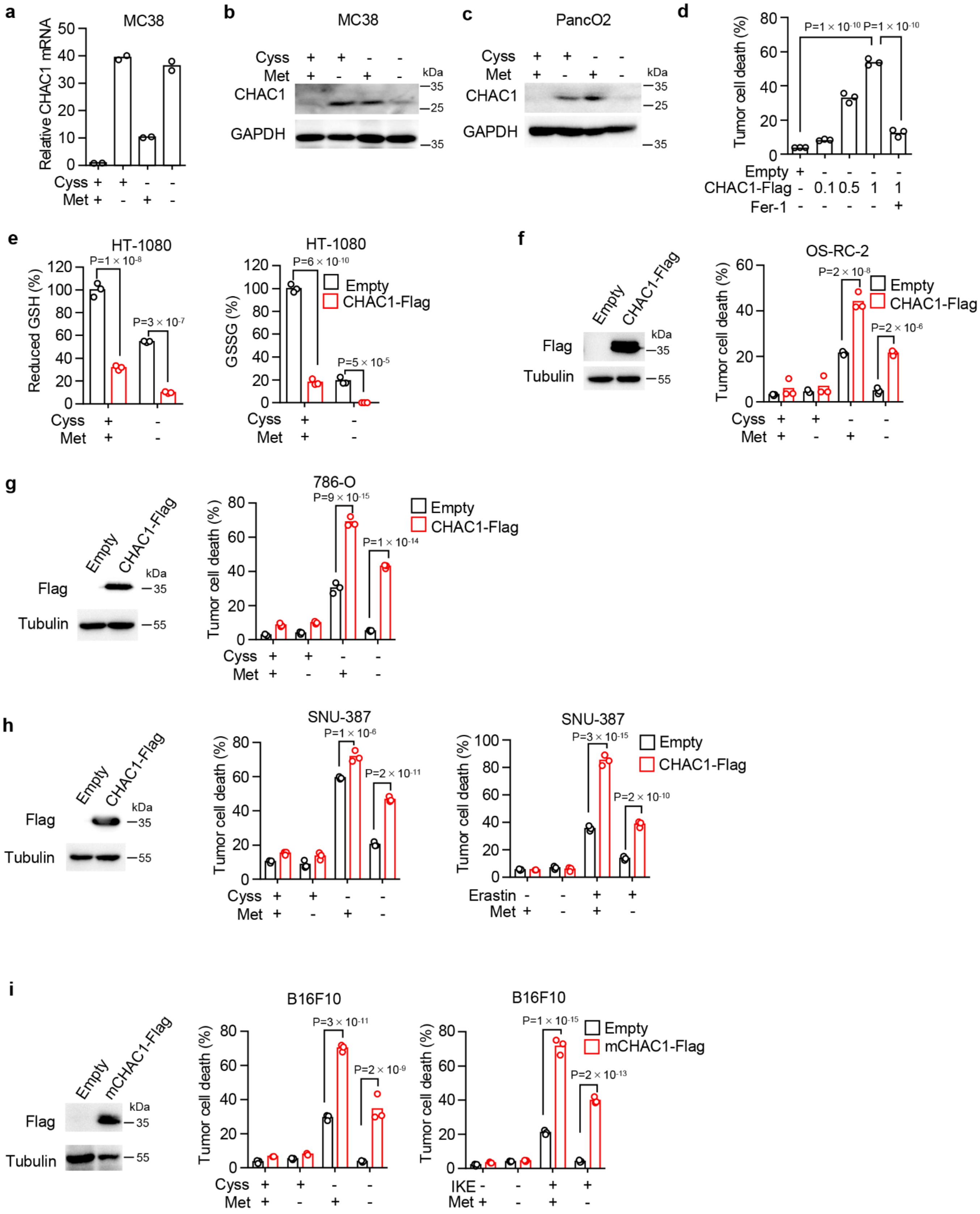
a
MC38
Relative CHAC1 mRNA
Cyss + + - -
Met + - + -
b
MC38
Cyss + + - -
Met + - + -
CHAC1
GAPDH
kDa
c
PancO2
Cyss + + - -
Met + - + -
CHAC1
GAPDH
kDa
d
Tumor cell death (%)
P=1 × 10-10 P=1 × 10-10
Empty + - - - -
CHAC1-Flag - 0.1 0.5 1 1
Fer-1 - - - - +
e
HT-1080
Reduced GSH (%)
P=1 × 10-8
P=3 × 10-7
Cyss + -
Met + -
HT-1080
GSSG (%)
P=6 × 10-10
P=5 × 10-5
Empty
CHAC1-Flag
Cyss + -
Met + -
f
Empty
CHAC1-Flag
Flag
Tubulin
kDa
OS-RC-2
Tumor cell death (%)
P=2 × 10-8
P=2 × 10-6
Empty
CHAC1-Flag
Cyss + + - -
Met + - + -
g
Empty
CHAC1-Flag
Flag
Tubulin
kDa
786-O
Tumor cell death (%)
P=9 × 10-15
P=1 × 10-14
Empty
CHAC1-Flag
Cyss + + - -
Met + - + -
h
Empty
CHAC1-Flag
Flag
Tubulin
kDa
SNU-387
Tumor cell death (%)
P=1 × 10-6
P=2 × 10-11
Cyss + + - -
Met + - + -
SNU-387
Tumor cell death (%)
P=3 × 10-15
P=2 × 10-10
Empty
CHAC1-Flag
Erastin - - + +
Met + - + -
i
Empty
mCHAC1-Flag
Flag
Tubulin
kDa
B16F10
Tumor cell death (%)
P=3 × 10-11
P=2 × 10-9
Cyss + + - -
Met + - + -
B16F10
Tumor cell death (%)
P=1 × 10-15
P=2 × 10-13
Empty
mCHAC1-Flag
IKE - - + +
Met + - + -

Supplementary Figure 4. Prolonged methionine deficiency inhibits ferroptosis by interrupting CHAC1 synthesis induced by cystine deprivation.

a, b, Relative mRNA (**a**) and protein (**b**) of mouse CHAC1 in MC38 cells treated with cystine withdrawal plus methionine co-withdrawal for 10 h.

c, Immunoblot of CHAC1 in PancO2 cells treated with cystine withdrawal plus methionine co-withdrawal for 10 h.

d, Cell death of HT-1080 cells after 20 hours of transient transfection with 1 μg empty plasmid or 0.1, 0.5 or 1 μg CHAC1-Flag expressing plasmid in the presence of Fer-1 (10 μM).

e, Reduced GSH and oxidized GSSG in CHAC1-Flag expressing HT-1080 cells treated with cystine withdrawal plus methionine co-withdrawal for 6 h.

f, g, OS-RC-2 (**f**) or 786-O (**g**) cells transduced with control (empty) or CHAC1-Flag expressing lentivirus. Immunoblot of Flag-tagged CHAC1 in these cells. Cells were treated with cystine withdrawal plus methionine co-withdrawal for 12 h (**f**) or 16 h (**g**), and the percentages of dead cells was measured.

h, Immunoblot of Flag-tagged CHAC1 in SNU-387 cells transduced with control (empty) or CHAC1-Flag expressing lentivirus. Cells were treated with cystine withdrawal or 10 μM erastin plus methionine co-withdrawal for 15 h or 17 h, and the percentages of dead cells was measured.

i, Immunoblot of Flag-tagged mouse CHAC1 in B16F10 cells transduced with control (empty) or mCHAC1-Flag expressing lentivirus. Cell death of these cells treated with cystine withdrawal or 0.5 μM IKE plus methionine co-withdrawal for 18 h.

The experiment was repeated twice with similar results (**c**). n = 3 biological replicates and P values are determined by one-way ANOVA (**d**) or two-way ANOVA (**e- i**).

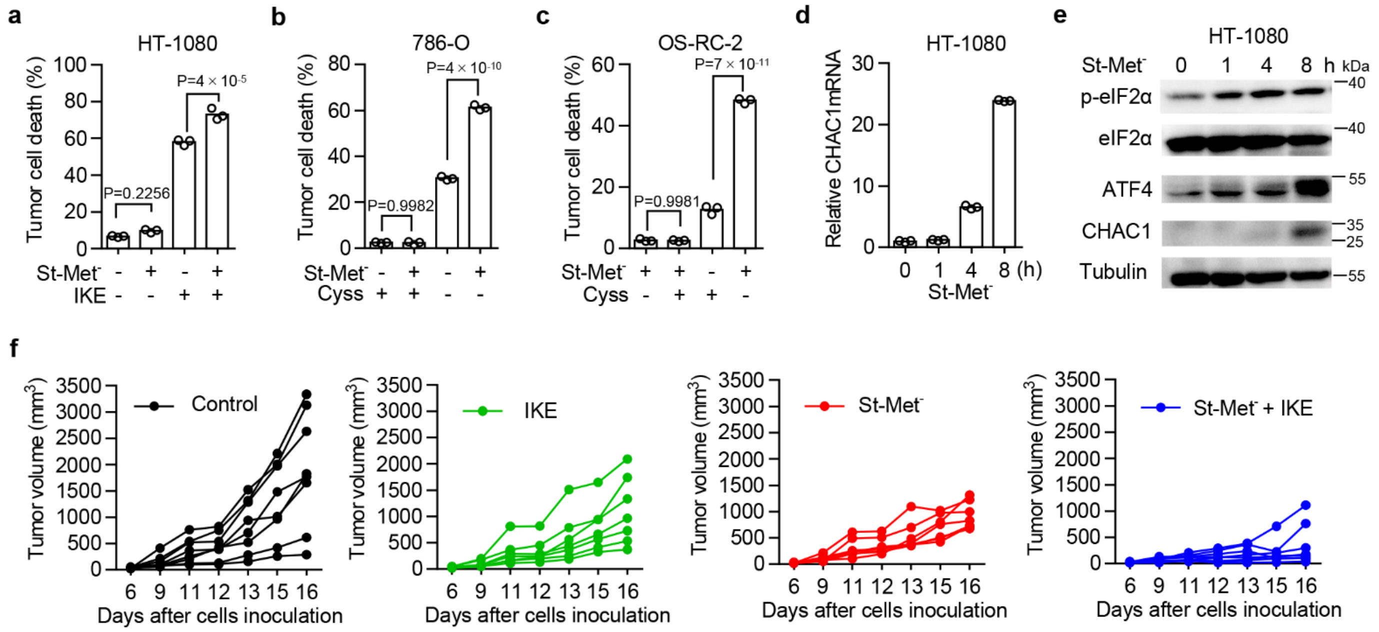

Supplementary Figure 5. Short-term methionine starvation promotes cystine deprivation-induced ferroptosis

a- c, Cell death of HT-1080 (**a**), 786-O (**b**) and OS-RC-2 (**c**) cells pre-treated by St-Met$^-$ for 4 h (**a**) or 2 h (**b, c**), and followed by IKE treatment (0.3 μM) for another 28 h (**a**) or by cystine deprivation for another 20 h (**b**) or 24 h (**c**). n = 3 biological replicates and P values are determined by one-way ANOVA.

d, Relative mRNA level of CHAC1 in HT-1080 cells treated by St-Met$^-$ for 1- 8 h.

e, Immunoblots of phosphorylated eIF2α (p-eIF2α), total eIF2α, ATF4 and CHAC1 in HT-1080 cells treated by St-Met$^-$ for 1- 8 h. The experiment was repeated twice with similar results.

f, B16F10 tumour growth in individual mice that were treated by control vehicle, IKE, dietary methionine intermittent starvation or their combination shown in Fig. 5i.

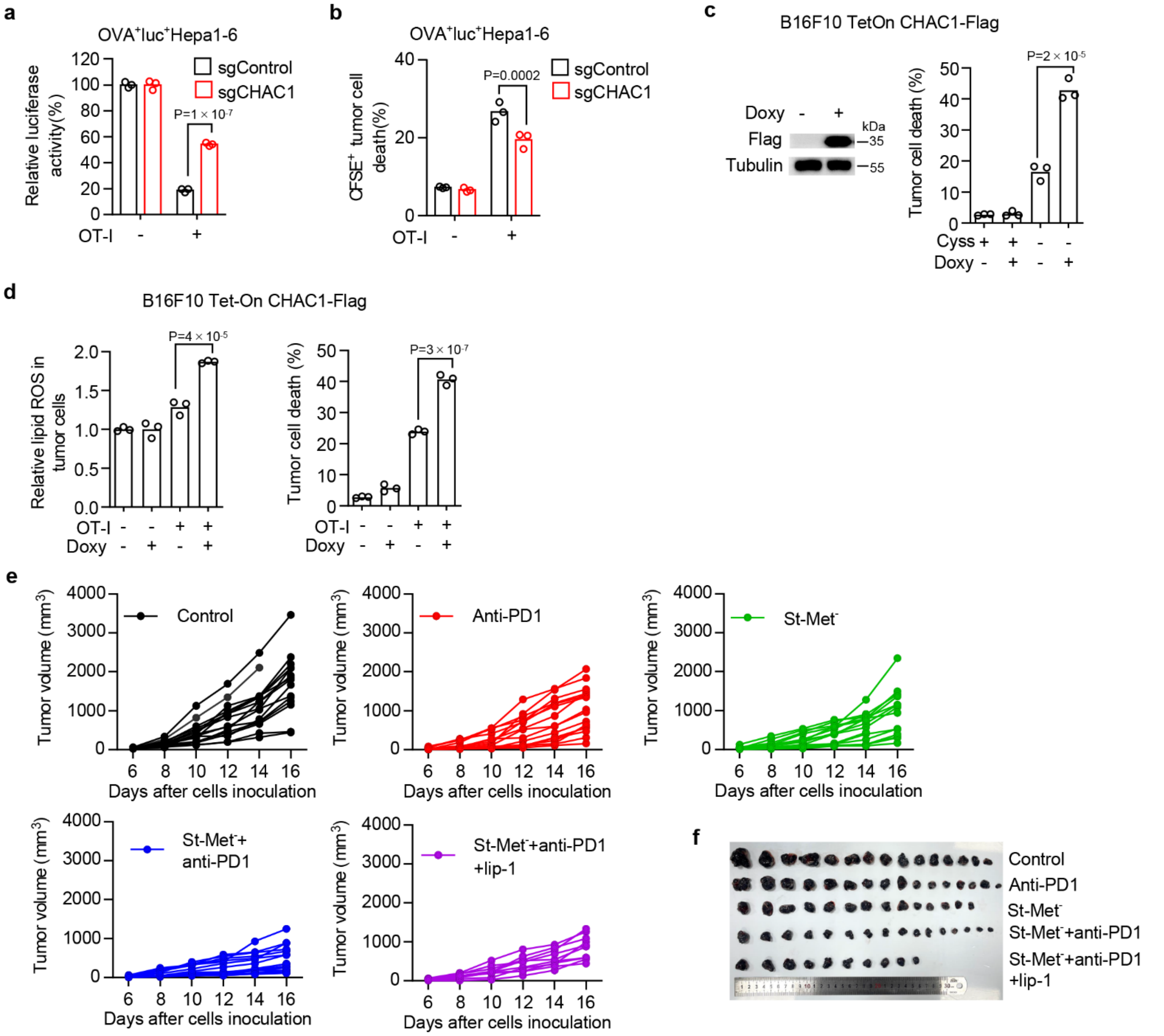

Supplementary Figure 6. Short-term methionine starvation sensitizes tumor against CTL-mediated killing and PD-1 blockade therapy

a, b, Control (sgControl) or CHAC1 deficient (sgCHAC1) OVA^+Luc^+ Hepa1-6 cells were co-cultured with OT-I cells (tumor: T= 1: 4) for 2 days. Luciferase activity (**a**) or the percentage of $CFSE^+$ $7\text{-}AAD^+$ tumor cells (**b**) in the co-culture system was quantified.

c, Immunoblot of Flag-tagged CHAC1 in B16F10 cells transfected with a Tet-On-inducible CHAC1-Flag expressing plasmid. Cells were treated with or without Doxy (0.5 μg/mL) for 48 h.

d, Tet-On-inducible CHAC1-Flag expressing B16F10 cells were pretreated with or without Doxy for 36 hours, pulsed with $OVA_{257-264}$ peptide and then co-cultured with OT-I cells (tumor: T= 1: 8). Lipid ROS level (left) or cell death (right) in tumor cells was quantified.

e, f, Individual tumor growth in mice treated by control vehicle, anti-PD-1 antibody, $St\text{-}Met^-$, the combination of $St\text{-}Met^-$ plus anti-PD-1 antibody or St-Met- + anti-PD1+ liproxstatin-1 shown in Figure

6e. Subcutaneous tumors were surgically removed and presented (**f**).

n = 3 biological replicates and P values are determined by one-way ANOVA (**c, d**) or two-way ANOVA (**a, b**).

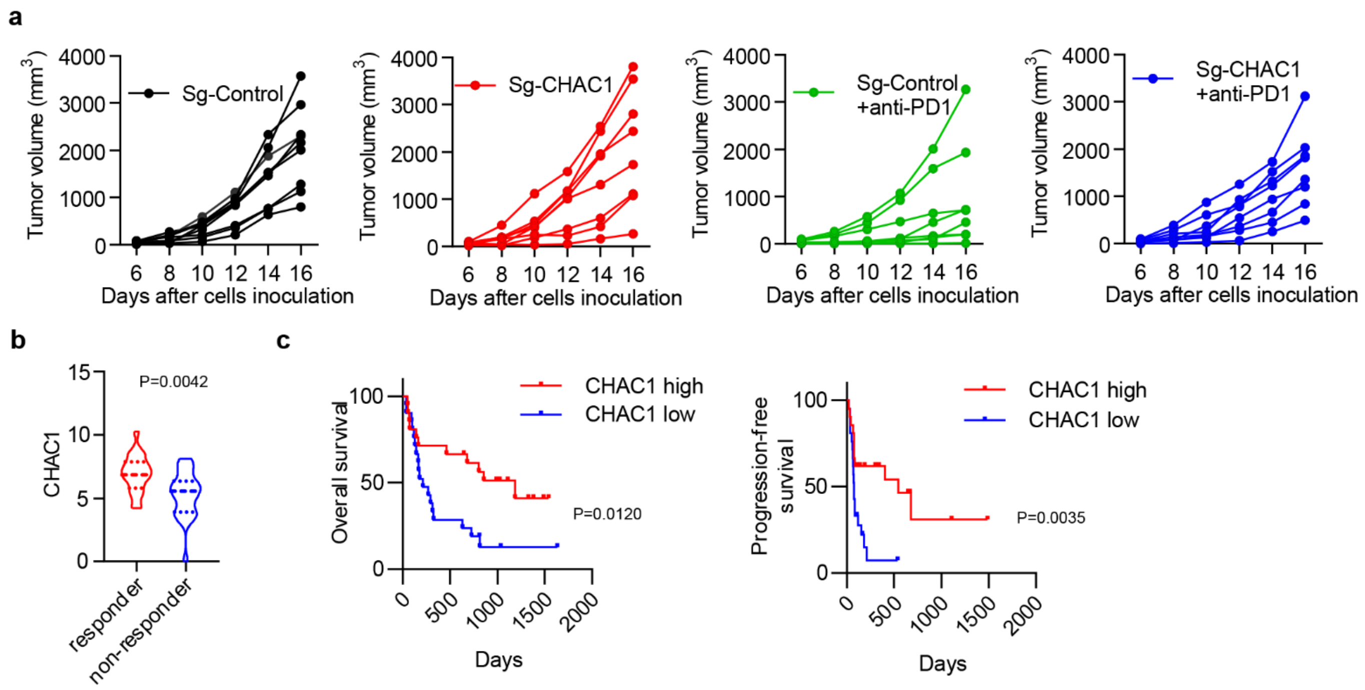

Supplementary Figure 7. Tumor CHAC1 contributes to the responsiveness of checkpoint blockade.

a, CHAC1 wildtype (sg-Control) or deficient (sg-CHAC1) B16F10 tumors bearing mice were treated by anti-PD-1 antibody shown in Fig. 7a. Individual tumor growth was monitored over time.

b, c, Violin plot comparing tumoral CHAC1 expression between responders and non-responders of melanoma patients who received anti-CTLA-4 therapy (**b**). Kaplan-Meier plots of overall survival (**c**, **left**) and progression-free survival (**c**, **right**) for these patients whose tumors demonstrate high vs low expression of CHAC1. P values are determined by two-sided Mann-Whitney test (**b**) or log-rank test (**c**).

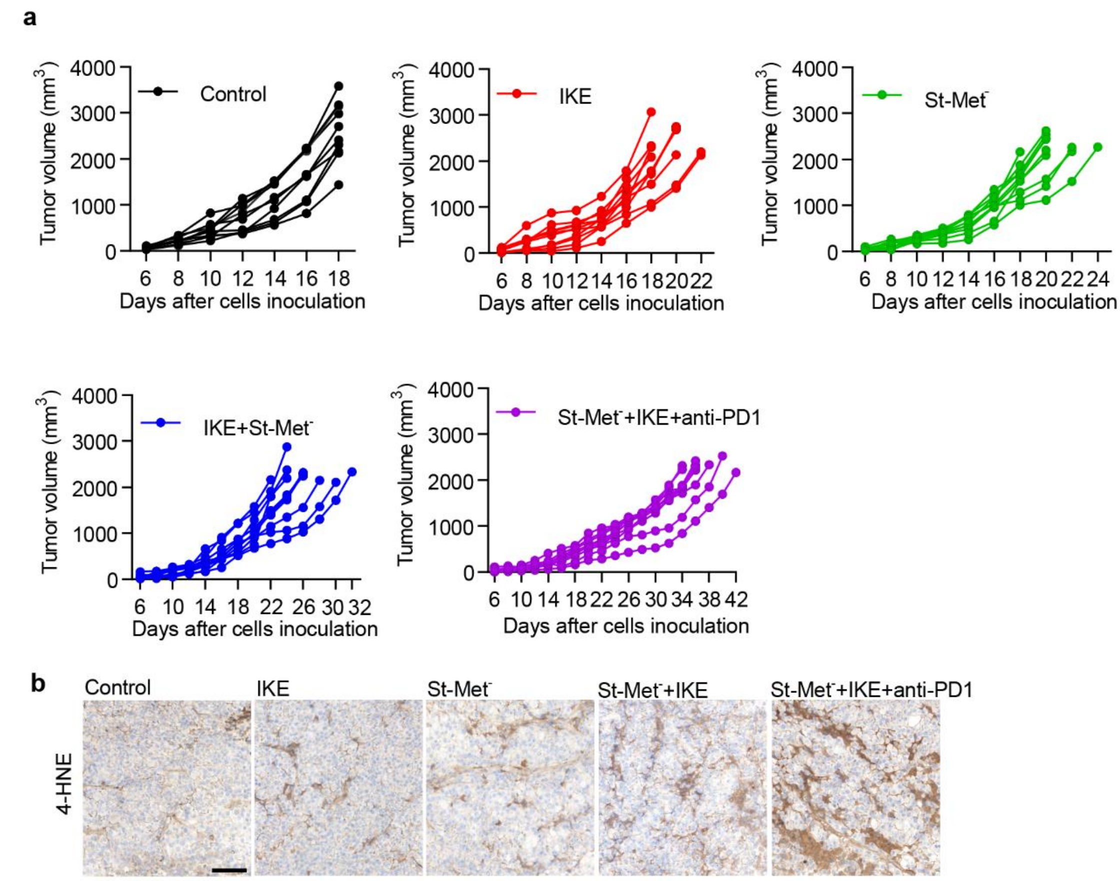

Supplementary Figure 8. Triple combination has the most potent antitumor efficacy.

a, B16F10 tumor-bearing mice were fed with Met^{+} diet (control) and treated with IKE (20 mg/kg) (IKE), or fed with Met^{-} diet intermittently ($St\text{-}Met^{-}$) and treated with IKE (20 mg/kg) ($St\text{-}Met^{-}$ + IKE) or IKE plus anti-PD-1 antibody (100 μg/mouse) ($St\text{-}Met^{-}$ + IKE + anti-PD1), n = 10 mice per group. Individual tumor growth from different groups was monitored over time. **b**, Images of immunohistochemistry for 4HNE in tumor tissue slides; scale bars,100 μm.

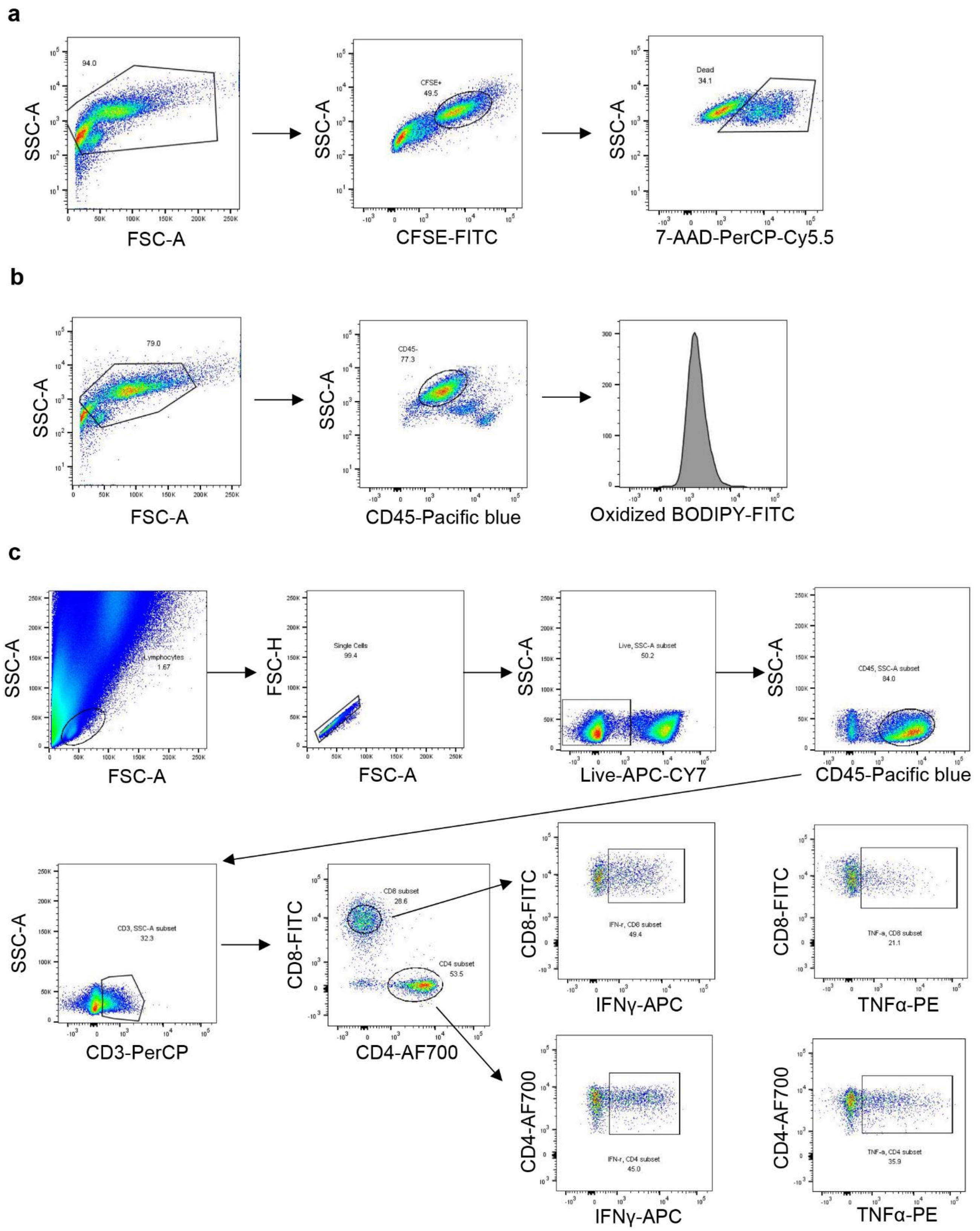

Supplementary Figure 9. Flow cytometry gating strategies for analyzing cell death, lipid peroxidation and tumor-infiltrating lymphocytes.

a, Flow cytometry gating strategy for cell death of $CFSE^+$ tumor cells in the co-culture system containing tumor cells and $CD8^+$ T cells, corresponding to the data shown in Figure 6b, e and Supplementary Figs. 6b, d.

b, Flow cytometry gating strategy for lipid peroxidation analysis in $CD45^-$ tumor cells from the co-culture of tumor and $CD8^+$ T cells, corresponding to the data shown in Figure 6c, f and Supplementary Fig. 6d (left).

c, Flow cytometry gating strategy for tumor-infiltrating lymphocytes isolated from subcutaneous B16F10 tumors, corresponding to the data shown in Figure 6n and 7b.

Supplementary Tables

Supplementary Table 1. Diet composition of methionine free diet

**Diet type**	Met^- diet		Met^+ diet	
Ingredient	gm	kcal	gm	kcal
L-Methionine	0	0	3	12
L-Alanine	3.5	14	3.5	14
L-Arginine	12.1	48.4	12.1	48.4
L-Asparagine-H_2O	6	24	6	24
L-Aspartate	3.5	14	3.5	14
L-Cystine	3.5	14	3.5	14
L-Glutamine	40	160	40	160
Glycine	23.3	93.2	23.3	93.2
L-Histidine-HCl-H_2O	4.5	18	4.5	18
L-Isoleucine	8.2	32.8	8.2	32.8
L-Leucine	11.1	44.4	11.1	44.4
L-Lysine-HCl	18	72	18	72
L-Phenylalanine	7.5	30	7.5	30
L-Proline	3.5	14	3.5	14
L-Serine	3.5	14	3.5	14
L-Threonine	8.2	32.8	8.2	32.8
L-Tryptophan	1.8	7.2	1.8	7.2
L-Tyrosine	5	20	5	20
L-Valine	8.2	32.8	8.2	32.8
Sucrose	455.3	1821.2	452.3	1809.2
Corn Starch	150	600	150	600
Maltodextrin 10	50	200	50	200
Cellulose	30	0	30	0
Corn Oil	100	900	100	900
Mineral Mix S10001	35	0	35	0
Sodium Bicarbonate	7.5	0	7.5	0
Vitamin Mix V10001	10	40	10	40
Choline Bitrartrate	2	0	2	0
Total	1011	4247	1011	4247
