# Supplementary material for: Intermittent dietary methionine deprivation facilitates tumoral ferroptosis and synergizes with checkpoint blockade
Source: Nat Commun. 2023 Aug 8;14:4758. doi: 10.1038/s41467-023-40518-0 (PMC10409767; doi:10.1038/s41467-023-40518-0)
Supplement: Supplementary file 3 — Reporting Summary [file 41467_2023_40518_MOESM3_ESM.pdf]

nature portfolio

Corresponding author(s): Weimin Wang

Last updated by author(s): Jun 27, 2023

Reporting Summary

Nature Portfolio wishes to improve the reproducibility of the work that we publish. This form provides structure for consistency and transparency in reporting. For further information on Nature Portfolio policies, see our Editorial Policies and the Editorial Policy Checklist.

Statistics

For all statistical analyses, confirm that the following items are present in the figure legend, table legend, main text, or Methods section.

n/a	Confirmed	
☐	☒	The exact sample size (*n*) for each experimental group/condition, given as a discrete number and unit of measurement
☐	☒	A statement on whether measurements were taken from distinct samples or whether the same sample was measured repeatedly
☐	☒	The statistical test(s) used AND whether they are one- or two-sided<br>*Only common tests should be described solely by name; describe more complex techniques in the Methods section.*
☒	☐	A description of all covariates tested
☐	☒	A description of any assumptions or corrections, such as tests of normality and adjustment for multiple comparisons
☐	☒	A full description of the statistical parameters including central tendency (e.g. means) or other basic estimates (e.g. regression coefficient) AND variation (e.g. standard deviation) or associated estimates of uncertainty (e.g. confidence intervals)
☐	☒	For null hypothesis testing, the test statistic (e.g. *F*, *t*, *r*) with confidence intervals, effect sizes, degrees of freedom and *P* value noted<br>*Give P values as exact values whenever suitable.*
☒	☐	For Bayesian analysis, information on the choice of priors and Markov chain Monte Carlo settings
☒	☐	For hierarchical and complex designs, identification of the appropriate level for tests and full reporting of outcomes
☒	☐	Estimates of effect sizes (e.g. Cohen's *d*, Pearson's *r*), indicating how they were calculated

Our web collection on statistics for biologists contains articles on many of the points above.

Software and code

Policy information about availability of computer code

Data collection	Flow cytometer BD Canto II (BD) , BD verse (BD) and Thermo Attune NxT (Thermo Fisher Scientific) were used to run cell samples and collect data for flow cytometry; QuantStudio® 3 Real-Time PCR System (Thermo Fisher Scientific) was used to run qPCR; ChemiDoc Imaging System (Tanon-5200Multi) was used for collecting data from immunoblotting experiments; Microplate reader (BioTek Synergy H1) was used for the assays requiring absorbency and luminescence quantification; Slide-scanning platform (3DHISTECH) was used for automated slide-scanning; OLYMPUS IX73 microscope was used for collecting microscopy images.
Data analysis	FlowJo software version 10 was used for FACS data analysis. QuantStudio Design and Analysis software v1.5.1 was for qPCR data analysis; Gen5 CHS 3.04 software was used for quantification of absorbency and luminescence data; Tanon AllDox-X software (v2.2.1) was used for analysis of immunoblotting results; CaseViewer2.4 software (3DHISTECH) was for analysis of IHC images; Cellsens Standard software for analysis of microscopy images; MarkerView1.3 and PeakView 2.2 (AB Sciex, Concord) were used to extract and analyze mess spectrometry data. GraphPad Prism version 8 and R were used for statistical analysis and P values calculation.

For manuscripts utilizing custom algorithms or software that are central to the research but not yet described in published literature, software must be made available to editors and reviewers. We strongly encourage code deposition in a community repository (e.g. GitHub). See the Nature Portfolio guidelines for submitting code & software for further information.

Data

Policy information about availability of data

All manuscripts must include a data availability statement. This statement should provide the following information, where applicable:

- Accession codes, unique identifiers, or web links for publicly available datasets
- A description of any restrictions on data availability
- For clinical datasets or third party data, please ensure that the statement adheres to our policy

The all data to support the conclusions in this manuscript can be found in the Article, Supplementary Information or Source Data file, which are provided alongside this paper. Metabolomics data corresponding to Figure 2a, 2b and Supplementary Fig. 2a generated in this study is provided in the Supplementary Data 1. Genomic data corresponding to Figure 3a is publicly available at the GEO with the accession number GSE60422 (https://www.ncbi.nlm.nih.gov/geo/query/acc.cgi?acc=GSE60422). Genomic data corresponding to Figure 7f, g and Supplementary Figs. 7b, c is publicly available at ENA under the accession number PRJEB23709 (https://www.ebi.ac.uk/ena/browser/view/PRJEB23709) and Cancer-Immu platform (http://bioinfo.vanderbilt.edu/database/Cancer-Immu/) or directly through phs000452.v2.p1 (https://www.ncbi.nlm.nih.gov/projects/gap/cgi-bin/study.cgi?study_id=phs000452.v2.p1). Source data are provided with this paper.

Research involving human participants, their data, or biological material

Policy information about studies with human participants or human data. See also policy information about sex, gender (identity/presentation), and sexual orientation and race, ethnicity and racism.

Reporting on sex and gender	N/A
Reporting on race, ethnicity, or other socially relevant groupings	N/A
Population characteristics	N/A
Recruitment	N/A
Ethics oversight	N/A

Note that full information on the approval of the study protocol must also be provided in the manuscript.

Field-specific reporting

Please select the one below that is the best fit for your research. If you are not sure, read the appropriate sections before making your selection.

☒ Life sciences ☐ Behavioural & social sciences ☐ Ecological, evolutionary & environmental sciences

For a reference copy of the document with all sections, see nature.com/documents/nr-reporting-summary-flat.pdf

Life sciences study design

All studies must disclose on these points even when the disclosure is negative.

Sample size	No statistical method was used to calculate sample size. Sample sizes were chosen based on our previous experiences (Nature. 2019;569 (7755):270-274; Cell. 2019;165(5):1092-105; Cancer Cell. 2022; S1535-6108(22)00036-8), which is sufficient to generate statistically significant results. For most of the in vitro experiments, at least three biological replicates were achieved for statistics. For in vivo experiments, a simple size of n = 7-16 mice per experimental group was used.
Data exclusions	No data was excluded.
Replication	All data from tissue culture were obtained with at least three biological replicates except for the experiments shown in Figure 4l and S4a, which were done by duplicate. The immunobloting experiments were repeated twice with similar results. For animal experiments shown in Figure 1k-m and 8d-e, 10 mice per group was used; for experiments shown in Figure 5g-k, 7a-b, 1c-e and 8a-c, at least 7 mice per group was used; and for experiments shown in Figure 6h-j, 16 mice per group was used. All replication attempts were successful.
Randomization	For in vivo experiments, age and sex matched animals were firstly inoculated with tumor cells and then randomly assigned into different treatment groups. The initial tumor burden was similar between the treatment and control groups. For in vitro experiments, cells were equally distributed into culture dishes and followed by different treatments with replications, no randomization was required.
Blinding	Researchers were blinded to LC-MS quantification of metabolites, isotopic tracing analysis and imaging analysis since the samples after treatment were labeled with code names by different investigators. All other experiments were performed in a non-blinded manner, because the experiments needed multiple treatments, including tumor cells and tumor-bearing mice treated by amino acid starvation, ferroptosis inducer and T cells or immunotherapy, and the different groups needed to be clearly labeled for the operation, data collection and analysis.

Reporting for specific materials, systems and methods

We require information from authors about some types of materials, experimental systems and methods used in many studies. Here, indicate whether each material, system or method listed is relevant to your study. If you are not sure if a list item applies to your research, read the appropriate section before selecting a response.

Materials & experimental systems

n/a	Involved in the study
☐	☒ Antibodies
☐	☒ Eukaryotic cell lines
☒	☐ Palaeontology and archaeology
☐	☒ Animals and other organisms
☒	☐ Clinical data
☒	☐ Dual use research of concern
☒	☐ Plants

Methods

n/a	Involved in the study
☒	☐ ChIP-seq
☐	☒ Flow cytometry
☒	☐ MRI-based neuroimaging

Antibodies

Antibodies used

Antibodies used for in vivo experiments: anti-mouse-PD-1 (Clone: RMP1-14, Cat#BE0146, Bio X Cell, 100µg/mouse).
Antibodies used for FACS:
anti-CD45 (Clone 30-F-11, Cat#103126, Pacific Blue, Biolegend, 1:100),
anti-CD3 (Clone 145-2C11, Cat#100325, PerCP, Biolegend, 1:100),
anti-CD90 (Clone 53-2.1, Cat#140319, BV510, Biolegend, 1:100),
anti-CD4 (Clone GK1.5, Cat#100430, Alexa Fluor 700, Biolegend, 1:100),
anti-CD8 (Clone 53-6.7, Cat#100706, FITC, Biolegend, 1:100),
anti-IFNγ (Clone XMG1.2, Cat#505810, APC, Biolegend, 1:100)
anti-TNFα (Clone MP6-XT22, Cat#506306, PE, Biolegend, 1:100).
Antibodies used for western blotting:
anti-CHAC1 (Proteintech, Cat#15207, 1:1000),
anti-human ATF4 (Proteintech, Cat#10835, 1:500),
anti-Flag (Sigma, Cat#1804, 1:2000),
anti-human eIF2α (CST, Cat#5324, 1:2000),
anti-human-EIF2S1 (phospho S51) (Abcam, Cat#ab32157, 1:1000),
anti-GAPDH (Proteintech, Cat#60004, 1:5000)
anti-alpha Tubulin (Proteintech, Cat#11224, 1:5000),
Goat anti-Rabbit IgG (H+L) Secondary Antibody, HRP (ThermoFisher Scientific, Cat#31460, 1:10000)
Goat anti-Mouse IgG (H+L) Secondary Antibody, HRP (ThermoFisher Scientific, Cat#31430, 1:10000).
Antibodies used for IHC: anti-4HNE (JaICA, Cat#HNEJ-2, 1:200).

Validation

The anti-mouse PD-1 antibody used in vivo and all antibodies used in FACS analysis targeting mouse proteins were well-recognized clones and validated by the manufacturers. The detailed information is provided through the following links. These antibodies have been routinely used in our laboratory. Primary antibodies targeting both human and mouse CHAC1 and human ATF4 were validated by the manufacturers, and they were further validated by our knockdown or knockout of the target gene by shRNA or CRISPR/Cas9, and verification of the loss of a band of the predicted molecular weight by western blot as shown in Figure 3f, j and Supplementary Fig. 3d,e,g. Primary antibodies targeting Flag-tag, human eIF2α, human EIF2S1 (phospho S51), human and mouse GAPDH and human and mouse Tubulin are validated by the manufacturers and their detailed information is provided through the following links. Antibody used for 4HNE IHC was verified by the manufacturer and the previous publication (PMID: 35922516). All validation statements are available on the antibody websites through the following links.
1. https://bioxcell.com/invivomab-anti-mouse-pd-1-cd279-be0146.
2. https://www.biolegend.com/en-us/products/pacific-blue-anti-mouse-cd45-antibody-3102.
3. https://www.biolegend.com/en-us/products/percp-anti-mouse-cd3epsilon-antibody-4190.
4. https://www.biolegend.com/en-us/products/brilliant-violet-510-anti-mouse-cd90-2-thy-1-2-antibody-7999.
5. https://www.biolegend.com/en-us/products/alexa-fluor-700-anti-mouse-cd4-antibody-3385.
6. https://www.biolegend.com/en-us/products/fitc-anti-mouse-cd8a-antibody-153.
7. https://www.biolegend.com/en-us/products/apc-anti-mouse-ifn-gamma-antibody-993.
8. https://www.biolegend.com/en-us/products/pe-anti-mouse-tnf-alpha-antibody-978.
9. https://www.ptgcn.com/products/CHAC1-Antibody-15207-1-AP.htm.
10. https://www.ptgcn.com/products/ATF4-Antibody-10835-1-AP.htm.
11. https://www.sigmaaldrich.cn/CN/zh/product/sigma/f1804.
12. https://www.cellsignal.cn/products/primary-antibodies/eif2a-d7d3-xp-rabbit-mab/5324.
13. https://www.abcam.com/products/primary-antibodies/eif2s1-phospho-s51-antibody-e90-ab32157.html.
14. https://www.ptgcn.com/products/GAPDH-Antibody-60004-1-Ig.htm.
15. https://www.ptgcn.com/products/TUBA1B-Antibody-11224-1-AP.htm.
16. https://qa1.thermofisher.cn/cn/zh/antibody/product/Goat-anti-Rabbit-IgG-H-L-Secondary-Antibody-Polyclonal/31460.

17. https://qa1.thermofisher.cn/cn/zh/antibody/product/Goat-anti-Mouse-IgG-H-L-Secondary-Antibody-Polyclonal/31430.
18. https://www.jaica.com/e/products_lipid_4hne_ab.html.

Eukaryotic cell lines

Policy information about cell lines and Sex and Gender in Research

Cell line source(s)	Human fibrosarcoma cell line HT-1080 (CCL-121), HEK293T (CRL-11268) and mouse melanoma cell line B16F10 (CRL-6475) were from the American Type Culture Collection (ATCC). Human hepatocarcinoma cell lines Hep3B (SCSP-5045), SNU387 (SCSP-5046) and SNU182 (SCSP-5047), Human renal carcinoma cell lines Caki-1 (SCSP-5064) and ACHN (SCSP-5063), mouse hepatocarcinoma cell line Hepa1-6 (SCSP-512) and mouse embryonic fibroblast cell line NIH/3T3 (SCSP-515) were from the China Center for Type Culture Collection (CCTCC). OS-RC-2 and 786-O cell lines are gifts from Dr. Xiangping Yang (Huazhong University of Science and Technology) who obtained them from CCTCC and ATCC. HT-29 originating from ATCC (HTB-38) are kindly provided by Dr. Zheng Wang (Huazhong University of Science and Technology) and PancO2 is a gift from Dr. Jun Zhao (Huazhong University of Science and Technology) who obtained from the Cell Resource Center, Peking Union Medical College. HT-1080, Hep3B and ACHN were cultured in MEM medium (Gibco, Thermo Fisher), Caki-1 was cultured in McCoy's 5A medium (Procell), NIH3T3 and PancO2 were cultured in DMEM medium (Gibco, ThermoFisher Scientific), and all other cells were cultured in RPMI medium (Gibco, ThermoFisher Scientific). All culture medium were supplemented with 10% FBS (Cegrogen Biotech).
Authentication	Hepa1-6 cell line was authenticated using the short tandem repeat analysis. It was done by a PCR-based strategy to target 17 STR loci and the result showed that the cell line matched with Hepa1-6 in Cellosaurus database. All other cell lines were not authenticated.
Mycoplasma contamination	All cell lines in our laboratory are routinely tested for mycoplasma contamination using a PCR-based method. And the cells used in this study are negative for mycoplasma.
Commonly misidentified lines (See ICLAC register)	No cell line used in this study is listed in ICLAC database.

Animals and other research organisms

Policy information about studies involving animals; ARRIVE guidelines recommended for reporting animal research, and Sex and Gender in Research

Laboratory animals	Wild type C57BL/6 mice, aged 6- 8 weeks, were obtained from Beijing Vital River Laboratory Animal Technology Co., Ltd. C57BL/6-Tg (TcraTcrb) 1100Mjb/J (OT-1 mice) were kindly provided by Dr. Ning Wu (Huazhong University of Science and Technology) and mice were bred and housed in the animal facility of Huazhong University of Science and Technology. Both female and male OT-I mice aged 8- 10 weeks were used in the experiments.<br>All animals were housed under specific pathogen-free conditions in groups of 5 mice per cage, and maintained in a humidity-controlled environment with a 12 h light/dark cycle at a temperature of 22-25°C.
Wild animals	The study did not involve wild animals.
Reporting on sex	For inoculation of Hepa1-6 tumors, 6 to 8 week-old male C57BL/6 mice were used; for all other tumor inoculation experiments, female mice were used.
Field-collected samples	The study did not involve samples collected from field.
Ethics oversight	Animal studies were conducted in accordance with the Institutional Animal Care and Use Committees and Institutional Review Board of School of Basic Medicine, Tongji Medical College, Huazhong University of Science and Technology.

Note that full information on the approval of the study protocol must also be provided in the manuscript.

Flow Cytometry

Plots

Confirm that:

- [x] The axis labels state the marker and fluorochrome used (e.g. CD4-FITC).
- [x] The axis scales are clearly visible. Include numbers along axes only for bottom left plot of group (a 'group' is an analysis of identical markers).
- [x] All plots are contour plots with outliers or pseudocolor plots.
- [x] A numerical value for number of cells or percentage (with statistics) is provided.

Methodology

Sample preparation	For cell death assays, tumor cells including the suspended dying cells were collected and resuspended in 200 µl PBS containing 1 µg/ml Propidium Iodide (PI) or 7-Aminoactinomycin D (7-AAD) for 10 minutes, and immediately run on a flow cytometer. PI or 7-AAD positive population was shown as the percentage of dead cells. For the co-culture of OVA+ tumor

cells and OT-I cells, 1 μM CFSE was used to label tumor cells first. After co-culture, all cells were harvested by trypsinization and stained with 7-AAD. Tumor cell death was analyzed by gating CFSE+ 7-AAD+ cell population.

For intracellular lipid peroxidation analysis, tumor cells were harvested by trypsinization, centrifugation and resuspended in 300 μl phosphate-buffered saline (PBS) containing 5 μM BODIPY 581/591 C11. Cells were incubated for 20 minutes at 37 °C in a tissue culture incubator, washed and resuspended in 200 μl PBS, then analyzed immediately on a flow cytometer. For the co-culture of OVA+ tumor cells and OT-I cells, supernatant was removed from co-culture system and the cells were harvested by trypsinization and stained with Pacific Blue anti-CD45 (30-F11) for 10 min at room temperature. And then cells were resuspended in PBS containing 5 μM BODIPY 581/591 C11 and incubated for 15 min at 37 °C. Cells were washed and analyzed immediately on flow cytometer.

To quantify T cells and their cytokines production, fresh tumor tissues were harvested, excised and ground into a single cell suspension. Lymphocytes were obtained by density gradient centrifugation with lymphocyte separation medium (MP Biomedicals) and stimulated in culture medium supplemented with PMA (10 ng/ml), Ionomycin (1 μg/ml), Brefeldin A (1: 1000) and Monensin (1: 1000) at 37°C for 5 hours. Cells were firstly stained in PBS containing Zombie NIR Dye (Biolegend) to identify dead cells. Anti-CD45 (30-F11), anti-CD3 (145-2C11), anti-CD90 (53-2.1), anti-CD4 (GK1.5) and anti-CD8 (53-6.7) were then added for surface staining. Then cells were washed and resuspended in 500 μl Fixation/perm solution (Biolegend) at room temperature (RT) for 30 minutes, and followed by staining with anti-IFNγ (XMG1.2) and anti-TNFα (MP6-XT22) for 20 minutes after washed with the Perm/Wash buffer (Biolegend). The cells were then washed and fixed in 4% formaldehyde (Biosharp).

Instrument

Flow cytometer BD canto II, BD verse or Thermo Attune NxT.

Software

FlowJo software version 10

Cell population abundance

No FACS-sorting was involved in the study.

Gating strategy

Tumor cells: The cells were gated on FSC-A/SSC-A basis. To analyze cell death or lipid peroxidaiton, CFSE positive or CD45 negative population was gated as tumor enriched. The percentage of 7AAD+ population or mean fluorescence intensity of ROS probe were analyzed.
Lymphoid cells: The cells were gated on FSC-A/SSC-A basis and single cells were gated on FSC-H/FSC-A basis. Zombie NIR Dye (Biolegend) was used to exclude dead cells. CD8+ T cells: CD45+/CD3+/CD8+/CD4-; CD4+ T cells: CD45+/CD3+/CD4+/CD8-. In CD8 and CD4 gate, the percentage of IFNg+ or TNF+ cells were analyzed.

☒ Tick this box to confirm that a figure exemplifying the gating strategy is provided in the Supplementary Information.
