# Supplementary material for: Intermittent dietary methionine deprivation facilitates tumoral ferroptosis and synergizes with checkpoint blockade
Source: Nat Commun. 2023 Aug 8;14:4758. doi: 10.1038/s41467-023-40518-0 (PMC10409767; doi:10.1038/s41467-023-40518-0)
Supplement: Supplementary file 4 — Description of Additional Supplementary Files [file 41467_2023_40518_MOESM4_ESM.docx]

Description of Additional Supplementary Files:

File Name: Supplementary Data 1

Description: Relative contents of intracellular metabolites that are identified by LC-MS in HT-1080 cells treated by cystine or methionine deprivation or their co-deprivation. Data provided as an Excel file.
